# Application of Hybrid Membrane Processes Coupling Separation and Biological or Chemical Reaction in Advanced Wastewater Treatment

**DOI:** 10.3390/membranes10100281

**Published:** 2020-10-13

**Authors:** Raffaele Molinari, Cristina Lavorato, Pietro Argurio

**Affiliations:** Department of Environmental Engineering, University of Calabria, via Pietro Bucci, Cubo 44/A, I-87036 Arcavacata di Rende (CS), Italy; cristina.lavorato@unical.it

**Keywords:** membrane technology, hybrid membrane processes, photocatalytic membrane reactors, liquid membranes, complexation-ultrafiltration, wastewater treatment

## Abstract

The rapid urbanization and water shortage impose an urgent need in improving sustainable water management without compromising the socioeconomic development all around the world. In this context, reclaimed wastewater has been recognized as a sustainable water management strategy since it represents an alternative water resource for non-potable or (indirect) potable use. The conventional wastewater remediation approaches for the removal of different emerging contaminants (pharmaceuticals, dyes, metal ions, etc.) are unable to remove/destroy them completely. Hybrid membrane processes (HMPs) are a powerful solution for removing emerging pollutants from wastewater. On this aspect, the present paper focused on HMPs obtained by the synergic coupling of biological and/or chemical reaction driven processes with membrane processes, giving a critical overview and particular emphasis on some case studies reported in the pertinent literature. By using these processes, a satisfactory quality of treated water can be achieved, permitting its sustainable reuse in the hydrologic cycle while minimizing environmental and economic impact.

## 1. Introduction

In the last decades, because of world population growth, urbanization, expansion of land used for agricultural use, and climate change, the global demand for freshwater has been significantly increased [1]. Besides, owing to desertification, the number of the regions and countries where the scarcity of freshwater represents a major challenge has been expanding in recent years, thus it significantly affects human activity and economic development. During the World Economic Forum held in 2019, the scarcity of fresh water was evidenced as one of the most limiting global risks in terms of potential impact over the next decade.

The interest on water reuse has increased all over the world [2,3]. For example, in recent years, the European Commission has been studying an appropriate regulation on water reuse [4]. To achieve water sustainability, reclaimed wastewater must be considered as an alternative water resource for irrigation [5], but also for ground or surface water recharge [6], because in most cases the wastewater treatment does not produce water for direct potable applications [7,8].

The conventional plants for wastewater treatment are not very effective in the removal of some emerging contaminants, such as pharmaceutical active compounds (e.g., antibiotics) and their metabolites [5]. These substances can affect the environment and human health, particularly if the effluents coming from the wastewater treatment plants are reused for irrigation purposes [9,10].

Other important water contaminants that deserve proper consideration are heavy metals, constituting an important health hazard [11,12,13]. For example, Cu^2+^ ions are essential nutrients, but long-term exposure to Cu^2+^ causes liver and kidney damage and produces DNA mutation [14]. Besides, copper toxicity has been implicated in various neurodegenerative disorders such as Wilson’s disease and Alzheimer’s disease. Traditionally, metal ions are separated/removed by processes such as precipitation, inorganic and polymeric adsorption, and reverse osmosis. These techniques produce water within international health standards, but they have some important drawbacks [15].

On these bases, the development of appropriate water treatment techniques, characterized by high performances and low environmental impact, to ensure a sustainable water management, represents a key point in the worldwide strategies [15].

With such a perspective, membrane technology can offer new opportunities in process design, rationalization, and optimization. Compared to traditional techniques of water treatments, the use of membrane processes has shown great potential in terms of increased water quality and separation selectivity, efficient materials recovery, low capital investment and operating costs, low energy consumption, constant temperature operation with no phase change, and process sustainability, especially when acceptable performance are not achievable with traditional treatments [1].

In recent years, many biological and physicochemical treatment methods have been investigated for the removal of emerging contaminants from wastewater. However, biologically based methods for wastewater treatment are not suitable for all wastewater compositions and the treatment efficiency is highly influenced by environmental conditions such as temperature, feed composition, and oxygen level [16,17]. Due to the deficiency of these methods, to completely remove these emerging contaminants, membrane processes, mainly microfiltration (MF) or ultrafiltration (UF), have been shown to produce a high quality permeate suitable to be directly discharged in compliance with legislation limits when coupled with/integrated into traditional treatment methods [18,19,20].

The coupling of biological and/or physicochemical treatment methods with membrane processes generates an important class of the so-called hybrid membrane processes (HMPs), which can offer the best solution in terms of separation efficiency and cost. In general, an HMP is a process that combines a membrane filtration unit with other processes/techniques, such as ion exchange, adsorption, catalytic ozonation, photocatalysis, coagulation and electrocoagulation, and humidification/dehumidification process [21]. It can also be a combination of different membrane operations (e.g., MF/UF-NF, UF-RO, etc.) in the same system with a conventional physical process. In the first case, an HMP combining separation and conversion (biological or chemical) is obtained while in the second case an HMP combining separation and physical treatment is obtained. These HMPs are useful “green” technologies, which improve the potentialities of classical treatment methods and those of membrane processes (separation at molecular level), giving a synergy for both technologies and thus minimizing environmental and economic impacts [22,23]. The membrane permits continuous operation in systems in which the removal of the pollutant and the production of high-quality filtered water simultaneously occur. Higher energy efficiency, modularity, and easy scale up are some other potential advantages of HMPs [24]. The listed advantages of HMPs are beneficial to a wide range of applications, including water and wastewater treatment, food and beverage production, pharmaceuticals, mineral processing, chemicals, fine chemicals, and bio-products.

In the present paper, among all the various HMPs application area, we focused on the wastewater treatment, which is one industrial area where membranes have been successfully applied [25]. Despite the fact that in the literature there are a lot of reviews on the applicability of HMPs in water and wastewater treatment [21,23,26], the wastewater remediation from emerging contaminants has not been properly considered. In particular, we describe the HMPs obtained by coupling membrane processes with biological or chemical reaction-driven processes. The following subcategories with some related applications are reported, as they seem particularly interesting in pollutants removal from wastewaters:

(i) HMPs obtained by coupling membrane processes with photocatalysis (PC);
(ii) HMPs obtained by coupling membrane processes with solvent extraction;
(iii) HMPs obtained by coupling UF membranes with water soluble complexants;
(iv) HMP obtained by coupling a photocatalytic membrane reactor and complexation–ultrafiltration (CP–UF) process;
(v) other hybrid membrane processes.

## 2. HMPs Obtained by Coupling Membrane Processes with Photocatalysis

Heterogeneous photocatalysis is an advanced oxidation process (AOP) that is based on the use of a photocatalyst, which is excited by light to generate the oxidizing/reducing species. Then, the photocatalyst is activated by a photonic mode, which replaces the thermal activation mode of the classical catalysis. The photocatalyst is practically a semiconductor, whose electronic structure is characterized by a conduction band (CB) and a valence band (VB). These bands are separated by a band gap of energy (Eg). When the photocatalyst is excited by photons with energy equal to or higher than their Eg, valence electrons (e^−^) are promoted from VB to CB, thus leaving a positive hole (h^+^) in the VB. Heterogeneous photocatalysis has been extensively studied since about five decades ago, when Fujishima and Honda [27] discovered the photocatalytic splitting of water on TiO_2_ electrodes. In particular, it has been largely studied for wastewater treatment by taking advantage of the total degradation to innocuous substances of organic and inorganic pollutants and by the removal of toxic metals. This process permits the user to operate under mild operating conditions (ambient temperature and pressure), requires few auxiliary additives, and results in the possibility to mineralize refractory, very toxic, and nonbiodegradable molecules.

In view of large-scale applications of photocatalysis, the confinement of the photocatalyst into the reaction environment has to be considered. On this aspect, the coupling of photocatalysis with a membrane separation represents a very promising approach. Photocatalytic membrane reactors (PMRs) can be defined as devices where photocatalysis and membrane separation are coupled to produce chemical transformations. This coupling produces a synergistic effect, improving the potentialities of classical photoreactors (PRs) and those of membrane processes. Indeed, the membrane permits conducting a continuous process in a system in which the photocatalytic reaction, the recovery of the photocatalyst, and the separation of the products from the treated effluent simultaneously occur.

In a PMR, the membrane can play many roles. The membrane is responsible for maintaining the photocatalyst in the reaction environment, making it important to choose a membrane with 100% photocatalyst rejection. Besides, the membrane is in charge of the function to reject the substrates and their intermediates into the reaction environment, thus avoiding their passage in the treated permeate effluent and controlling the residence time of the molecules to be degraded. The latter aspect is important to obtain the complete degradation (i.e., mineralization) of the pollutants.

PMRs have been reported in literature for the degradation of different compounds contained in aqueous media. Different reviews have been reported describing the application of PMRs in the treatments of different wastewaters [24,28,29]. In the present section, some examples will be summarized, deferring the reader to the aforementioned reviews for further information.

### 2.1. PMRs in the Treatment of Primary and Secondary Effluents

The effluents coming from conventional municipal wastewater treatment plants (the so-called primary effluent (PE) and secondary effluents (SE)) frequently contain different biological and organic pollutants. These substances are hazardous to both human health and the receiving aqueous ecosystems. Moreover, the presence of these pollutants into the treated effluent decreases public acceptance of reusing treated effluents for industrial, agricultural, and domestic uses. As a result of this, effective post-treatments are required to abate or at least reduce the contamination level of these treated effluents, to ensure their safe release or reuse [1].

On this topic, in 2016 Jiang and Choo [30] investigated the performance of a submerged PMR utilizing TiO_2_ in suspension as a photocatalyst in the removal of secondary effluent organic matter (SEOM) from a SE taken from the Shincheon Municipal Wastewater Treatment Plant (Daegu, Korea). Organic removal and fouling control were the main aspects taken into consideration. The investigated PMR was equipped with a hollow fiber MF membrane made of hydrophilized polyethylene with a surface area of 60 cm^2^ and a nominal pore size of 0.4 μm. A blacklight blue UV (ultraviolet) lamp, with an energy consumption of 6.5 W and a photon flux of 40 mW cm^−2^ at 365 nm, was used as irradiation source. The PMR was operated continuously under constant flux mode (50 L m^−1^ h^−1^). The results evidenced that the tested PMR permitted to achieve a good degradation (more than 60%) of SEOM present in the SE, while reducing membrane fouling propensity. The photocatalytic efficiency was reduced by the presence of particles in the SE, the presence of air bubbles, as well as the attachment of TiO_2_ particles onto the membrane. Application of bubbleless backpulsing (30 s every 1 h at a pressure of 0.05 MPa) permitted the researchers to obtain a better control of membrane fouling as well SEOM degradation than extensive aeration, while significantly reducing energy consumption.

**Figure 1:**
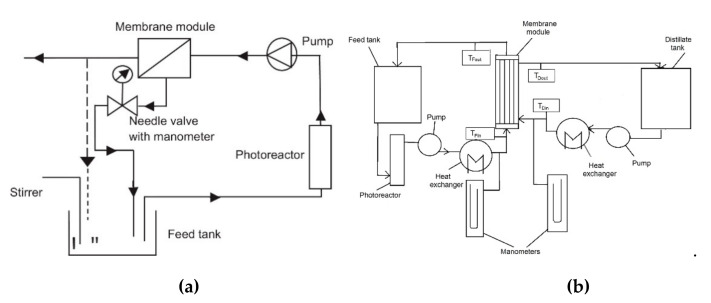
Schematic diagram utilizing ultrafiltration (UF) (**a**) and direct contact membrane distillation (DCMD) (**b**) [31].

On the same topic, Mozia et al. [31] tested two different PMRs (see Figure 1), obtained by coupling photocatalysis with ultrafiltration (UF) and direct contact membrane distillation (DCMD), for treating PE and SE coming from a municipal wastewater treatment plant. The performances of these two PMRs were compared with that one obtained by using UF and DCMD alone (i.e., without PC).

The results evidenced that in both the studied PMRs the permeate flux obtained by treating the PE was significantly lower than that one obtained with the SE. This behavior was attributed to the higher turbidity (75–110 vs. 1–4 NTU) and TOC (total organic carbon) concentration (57–85 vs. 10–19 mg L^−1^) of PE. The coupling of photocatalysis with membrane processes permitted researchers to increase the permeate flux only in the case of UF, compared to UF alone. This flux increment was in the range 25–38% by treating the PE, and 33% in the case of SE. This flux improvement was attributed to the formation of more porous cake layer compared to that one obtained by using UF alone, as well as to improved hydrophilicity of the membrane covered with TiO_2_. No influence of the presence of TiO_2_ on permeate flux was found by coupling photocatalysis and DCMD. In this PMR, no flux decline was observed by treating SE, while a 40–50% flux decline was observed by treating the PE. This trend was explained by considering the strong interactions between the hydrophobic polypropylene membrane and the hydrophobic contaminants. As a consequence, the formation of a dense fouling layer took place both on the membrane surface and inside the membrane pores, which contributed to the increase of both mass and thermal resistances. Permeate quality was higher in DCMD-PMR than in UF-PMR. Considering that TOC mineralization in the feed during 5 h of experiments was very low, this higher water quality was attributed to membrane separation rather than to the photocatalytic degradation.

Several investigations have shown that an important class of pollutants that are not completely removed during conventional municipal wastewater treatments is represented by pharmaceuticals and their metabolites [22,32]. These substances have been detected in the aquatic environment at concentrations up to μg L^−1^ [33,34]. Three different hybrid systems obtained by coupling ultrafiltration (UF) with UVC/H_2_O_2_, UVC/TiO_2_, and UVC (ultraviolet C light) were tested by Espindola et al. [35] in the treatment of a secondary effluent (SE) coming from a municipal wastewater treatment plant spiked with 5 mg L^−1^ of oxytetracycline (OTC). The effect of H_2_O_2_ concentration (varied in the range 30–120 mg L^−1^) and of TiO_2_ loading (varied in the range 0.5–1.5 g L^−1^) were investigated in the UVC/H_2_O_2_-UF and UVC/TiO_2_-UF systems, respectively. The results evidenced that the permeate flux was significantly influenced by the oxidant concentration and the photocatalyst loading. A 100% OTC removal and a 49% mineralization were achieved in 5 h with 1.0 g L^−1^ of TiO_2_ in the UVC/TiO_2_-UF system. During the same time interval, a dissolved organic carbon (DOC) removal of 52% was obtained by using the UVC/H_2_O_2_-UF system with an H_2_O_2_ of 120 mg L^−1^. A similar permeate flux decrease (40%) with respect to pure water flux (111 L m^−2^ h^−1^) was observed in both systems, while a 60% flux decrease was observed with the UVC-UF system, due to its low mineralization efficiency.

### 2.2. PMRs in the Photodegradation of Dyes in Aqueous Media

Azo-dyes represent the most common dyes actually utilized in textile industries. During the dyeing process, an amount of colorants in the range of 10–50% of the initial one is lost to the environment. Dyes can significantly affect the receiving aquatic environment by reducing sunlight transmission through water. In addition, their abatement represents a relevant problem in the wastewater treatment processes because generally they are very stable toxic compounds. For this purpose, photocatalytic membrane reactors can be interesting technologies employed for chemical conversion of organic dyes into safe (nontoxic) or harmless compounds with minimum consumption of energy and reagents [24,27,36,37].

Various types of membranes have been studied to be applied in photocatalysis, such as inorganic and polymeric, under UV and visible light irradiations [24,27,36,37,38,39,40,41,42,43]. Moreover, the use of visible light in photocatalysis permits the use of renewable solar energy in view to obtain a sustainable process [24,27]. The advantage of coupling a photocatalyst under visible light and the separation process in a single unit is that it allows users to obtain a lower degradation of polymeric membranes elongating their lifetime.

The photocatalytic degradation of RB5 reactive dye was studied by Ashar et al. under artificial sunlight by using ZnO and Fe^3+^@ZnO nano discs in PMRs [44]. The results showed 88.89% of dye degradation by using ZnO/PMR and 98.34% by using Fe^3+^@ZnO PMR in 180 min. The photocatalytic activity of Fe^3+^@ZnO PMR gradually decreased after eight reaction times.

An interesting study to reduce the presence of synthetic dyes in water concerns the possibility to couple photocatalysis and ultrafiltration [39]. For this purpose, Athanasekou et al. prepared ceramic ultrafiltration (UF) membranes with deposition of various photocatalysts (TiO_2_, graphene oxide-TiO_2_ composites) based nanomaterials on the external and internal (pore) surface of UF mono-channel monoliths. The photocatalytic filtration experiments were carried out using methylene blue (MB) and methyl orange (MO) as azo-dye model pollutants, in a patented water purification device in continuous flow conditions under near-UV/vis and visible light irradiation.

The synergistic effect of membrane filtration and photocatalysis was also studied by Zhang et al. for the removal of rhodamine B [45]. They reported more than 60% of rhodamine B could be removed from water under visible light irradiation by using g-C_3_N_4_ quantum dots (QDs) assembled into TiO_2_ nanotube array (TNA) membranes to obtain a visible-light-driven g-C_3_N_4_/TNA membrane. Furthermore, this membrane-integrated process showed also antifouling ability towards Escherichia coli under visible light irradiation, with a permeate flux of two times higher than that of filtration alone.

The possibilities to coat semiconductors on optical fibers can be promising in water treatment. For example, Hu et al., studied an integrated system by using P-doped g-C_3_N_4_ (PCN) as photocatalyst, coated on an Al_2_O_3_ substrate with Al_2_O_3_ hollow fiber membrane module as a PMR [46]. The highest degradation activity for methylene blue (MB) removal under visible irradiation was obtained by using 10 wt. % of PCN. Moreover, 92% of the phenol was decomposed and mineralized in the PMR. Also, repeating tests for four times, the efficiency of MB removal was always greater than 90%.

Fouling is an important problem for membrane purification, for this reason various authors studied photocatalytic membranes with antifouling and self-cleaning ability [47,48,49,50]. For this purpose, Sun et al. [47] prepared magnetic TiO_2_@Ni particles (MNPs) arranged onto the polymeric polyether sulfone (PES) membrane surface. Experimental tests were carried out under UV light and sunlight obtaining optimal membrane flux recovery ratio (FRR) of 75.4%, 99.56%, 92.11%, and 98.26% for BSA (bovine serum albumine), YEF (yeast extract fermentation), SA (ammonium alginate), and HA (humic acid) solutions, after self-cleaning.

Graphene oxide (GO) has been recently used as photocatalyst by various authors [48,49,50]. TiO_2_-doped GO membranes were used in water filtration and photodegradation of pollutants under UV light also to improve the membrane fouling [48]. Instead, Liu et al. doped the surface of GO and titanate nanotubes (TNTs) with Ag nanoparticles (Ag/GO/TNT) [49]. They reported that 90% of MB could be degraded after 120 min irradiation under visible light with a flux of 34.7 L m^−2^ h^−1^.

Alyarnezhad et al. [37] investigated the degradation of MB under visible light irradiation (λ > 420 nm) by using four types of membranes prepared by using a nontoxic solvent and GO nanosheets as a metal-free catalyst to photoactivate the membrane. The incorporation of GO enhanced the mechanical strength of the prepared membranes and their wettability. Moreover, the presence of one hydration layer on the membrane surface limited the fouling, decreasing the attachment of pollutants and microorganisms. A good photocatalytic performance (dye removal efficiency of 83.5%) for the degradation of MB^+^ under simulated solar light irradiation was obtained by using the membrane sample indicated by the authors as M8. This sample was prepared with a dope solution containing the following components: PVDF (polyvinylidene difluoride, 13 wt. %), PVP (polyvinyl pyrrolidone, 3 wt. %), PEG (polyethylene glycol, 24 wt. %), TEP (triethyl phosphate, 59.875 wt. %), and GO (0.125 wt. %) in the casting solution, and exposed for 2.5 min to moisture during the VIPS (vapour-induced phase separation) step.

**Figure 2:**
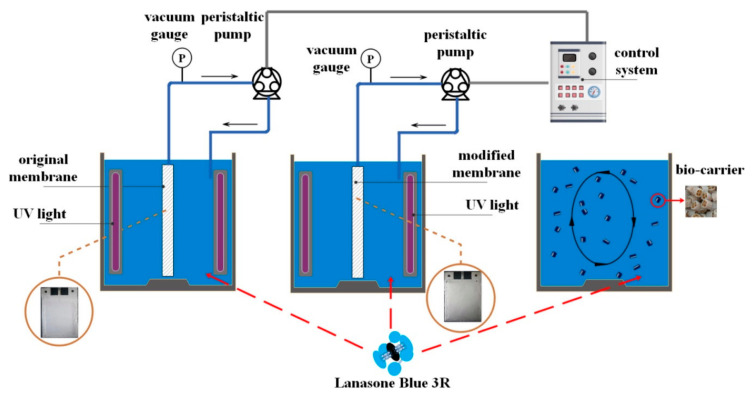
Schematic diagram of the cross-flow membrane filtration system [50].

Another type of membrane that couples the photocatalytic activity and mitigation of membrane fouling in a membrane reactor is a polyvinylidene fluoride flat sheet membrane-coated 3D TiO_2_/poly (sodium styrenesulfonate) TiO_2_/poly (sodium styrenesulfonate) (TiO_2_/PSS)_7_ fabricated via dip-coating layer-by-layer (LbL) assembly [50]. Cationic TiO_2_ and anionic PSS were alternately stacked on the support membrane via electrostatic interactions. The membrane reactor setup, showed in Figure 2, consisted of two 5 L plexiglas reactors with original membrane (OM) and the modified membrane (MM) (TiO_2_/PSS)_7_ and bed biofilm reactor (MBBR). The flux (300 L m^−2^ h^−1^) was maintained at a constant by adjusting the rotation rate of the peristaltic pump. The membrane surfaces were irradiated by two UV lamps (385 nm) placed on both sides of the original membrane and the modified membrane. Degradation experiments demonstrated that the MM had a self-cleaning ability, which was assisted by photocatalysis. MM (TiO_2_/PSS)_7_ was selected to investigate the self-cleaning and reusability of membrane and to demonstrate the photocatalytic activity on Lanasol Blue 3R (LB) degradation. Before each cycle, this membrane was irradiated with a UV lamp for 120 min to remove the LB adsorbed on the surface. The results showed a removal rate of LB up to 91.42% with high removal efficacy also after five cycles. The original PVDF membrane and TiO_2_/PSS-MM both exhibited a decreased flux after BSA filtration, but the irradiation with UV lamp for 8 h allowed researchers to obtain a flux of TiO_2_/PSS MM rose near to prefouling levels. In particular, (TiO_2_/PSS) exhibited excellent self-cleaning abilities (the recovery rate was up to 95.41%). The SEM images showed a clear surface of MM after self-cleaning. The authors reported a schematic mechanism: (i) the immobilization of TiO_2_ on the surface and membrane pores allowed the contact between TiO_2_ and foulants, (ii) TiO_2_ under UV light produced active free radicals such as **·**OH, (iii) degradation of the fouling layer formed during BSA filtration.

### 2.3. PMRs in the Photodegradation of Pharmaceuticals in Aqueous Media

The growing increase of environmental pollutants, such as pharmaceutical products, require the improvement of traditional wastewater treatments [24,27,51]. In the last few years, scientists have developed new materials that can be promising in water treatment, such as magnetic materials in photocatalytic composites and semiconductors coated on optical fibers for their adsorptive abilities towards pharmaceuticals for wastewater treatment [51]. Indeed, magnetic composites can facilitate the removal of the photocatalysts from water. For example, magnetic FeNi_3_/SiO_2_/CuS [52] can be used to remove tetracycline from wastewater, while to remove amoxicillin magnetic fluorinated mesoporous graphitic carbon nitride [53] and a magnetic TiO_2_-GO-Fe_3_O_4_ [54] can be used. Also, the immobilization of semiconductors on optical fibers enhanced their recovery [51].

Sulfamethoxazole and ibuprofen can be degraded by a composite semiconductor TiO_2_-rGO coated on optical fibers [55]. The presence of bisphenol A (BPA) in various water sources is dangerous because it can cause numerous adverse effects in humans. Kamaludin et al. [56] studied the removal of BPA from water under visible light by using a hollow fiber membrane. The authors reported the preparation of a photocatalytic dual-layer hollow fiber (DLHF) membrane fabricated via co-spinning phase inversion. N-doped TiO_2_ showed good photocatalytic activity under ultraviolet and visible irradiation with a band gap of 2.64 eV. DLHF membranes showed 90% BPA removal under UV light irradiation, while N-doped TiO_2_ DLHF removed 81.6% of BPA under visible light irradiation.

Another system, used for the degradation of methylene blue (MB) and chlorhexidine digluconate (CHD), consists of TiO_2_ nanoparticles immobilized on polymeric commercial hollow fiber (HF) ultrafiltration membranes. Chakraborty et al. [57] reported that a degradation of more than 30% and 40% of MB and CHD, respectively, was achieved under filtration conditions with simulated solar light radiation.

**Figure 3:**
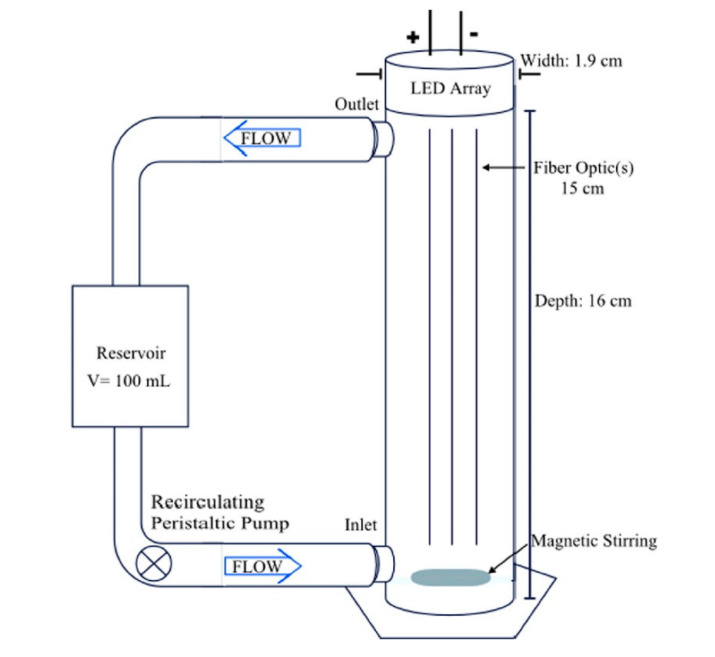
Flow-through optical fiber (OF)/LED reactor design [58].

Tugaoen et al. [58] designed and operated an optical fiber/LED (OF/LED) recirculating reactor system to remove parachlorobenzoic acid (pCBA), a model compound in simulated drinking water treatment. In Figure 3, the TiO_2_/optical fiber flow reactor constituted by a near clear PVC (polyvinyl chloride) cylinder (Harrington Plastics), with an inner diameter of 1.9 cm and a total length of 18 cm, is schematized. A peristaltic pump was used to circulate the solutions at 5 mL min^−1^ containing 0.1 mM of pCBA at pH 4.0. The coated optical fibers/LED couple (OF/LED) were inserted into the reactor at the distance of 1 cm. Three configuration (OF/LED) units, connected to a single LED source, were studied: (1) an individual fiber, (2) a bundle of three fibers, and (3) a bundle of fifteen fibers. Moreover, the number of OF/LED units, inside the reactor, varied from one to five. Highest kinetics were achieved for 1:1 coupling using five OF/5 LEDs. Contrariwise, with this configuration the lowest quantum yield (Φ) and the highest electrical energy per order (EEO) was obtained because many of the emitted photons were not utilized in the photocatalytic reactions of interest. Instead, when a single LED was coupled to bundled optical fibers, the available photoactive surface area increased. With fixed photon flux, pCBA oxidation increased with increasing the number of optical fibers according to all parameters of interest: kinetics, Φ, and EEO.

**Figure 4:**
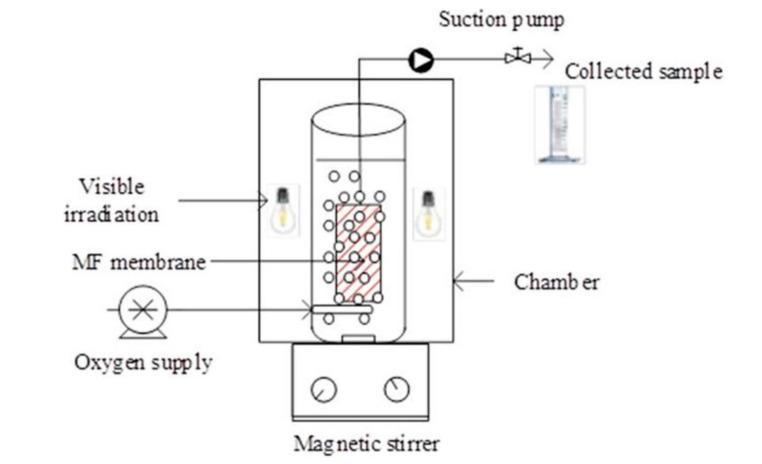
Submerged photocatalytic membrane reactor (SMPR) reactor set up [59].

An important pharmaceutical pollutant, usually employed by people for their anti-inflammatory activity, is Diclofenac (DCF). Because it is hardly biodegradable [59], a more efficient method to remove it from wastewater must be used. Very recently, Nguyen et al. [59] studied the photocatalytic activity of Visible/N-doped TiO_2_ and Visible/N-doped TiO_2_/H_2_O_2_ (with H_2_O_2_ addition to the reaction environment). Photocatalytic experimental tests were carried out in a submerged photocatalytic membrane reactor (SMPR) with suspended photocatalyst. The efficiency of organic removal was enhanced by using hydroxyl peroxide (H_2_O_2_). Moreover, N was more effective than other dopants (C, S, P) in narrowing the optical bandgap of TiO_2_ because of closing energy between N 2p state and O 2p state. The SMPR, Figure 4, was a cylindrical photoreactor (volume of 2 L) with an immersed tube of ceramic MF membrane and, around the reactor, five visible lamps of 50 W (420–720 nm). The membrane was connected to a suction pump to collect the water sample. The oxygen was continuously feed under the UF membrane. The experimental data showed a pseudo-first-order kinetic model. The results indicated that the addition of H_2_O_2_ improved the system performance while the efficiency of the process decreased by using a higher initial concentration.

The release of antibiotics in wastewater, such as amoxicillin (AMX), can have dangerous effects on the aquatic and terrestrial organisms due to their persistence in the environment. For this purpose Bergamonti et al. [60], studied TiO_2_/CS (TiO_2_/chitosan) for the amoxicillin photodegradation under UV/Vis irradiation. TiO_2_ chitosan scaffolds with photocatalytic activity for wastewater remediation were prepared by 3D printing using commercial P25-TiO_2_.

Another system consisted of a tube-in-tube membrane reactor, with hydrogen peroxide addition. The reactor was used for the oxidation of four pharmaceuticals: paracetamol (PCT), furosemide (FRS), nimesulide (NMD), and diazepam (DZP), in a continuous-mode operation [61]. This membrane reactor was used for photochemical and photocatalytic processes, driven by UVA (ultraviolet A light) or UVC photons. Lumbaque et al. performed photocatalytic tests using synthetic (SWW) and real (urban wastewater after secondary treatment) (UWW) matrices, both with the pharmaceutical mix solution of 200 μg L^−1^ of each. The highest removal percentage for each pharmaceutical, PCT (27.4%), FRS (35.0%), NMD (24.2%), and DZP (30.0%), in SWW was obtained at steady-state regime of the UVC/H_2_O_2_/TiO_2_ system, with radial H_2_O_2_ addition (20 mg L^−1^). Comparing the UWW and SWW matrices, a decrease in pharmaceuticals elimination was observed for UWW (PCT—11.5%, FRS—20.3%, NMD—8.2%, and DZP—12.6%).

## 3. HMPs Obtained by Coupling Membrane Processes with Solvent Extraction

### 3.1. Liquid Membrane (LM)-Based Operations

Selective separation of organic and inorganic compounds from different matrices is a critical issue in the chemical industry. Several conventional methods are commonly used for this purpose, including solvent extraction, chemical precipitation, ion exchange, adsorption, coagulation–flocculation, flotation, electrochemical methods, crystallization, and fractional distillation. These techniques generally meet legislation requirements, but they have some important drawbacks. For example, solvent extraction has the main drawback of using large quantities of organic phase, especially when processing diluted solutions, thus producing a large amount of waste solvents. Besides, there is also the risk associated to the use of inflammable and harmful solvents. Precipitation is a very simple technique, but it is unselective and produces a large amount of sludge containing contaminants and residuals of the precipitating agents. Adsorption and ion exchange are discontinuous, because of the regeneration necessity, which negatively affects process economy.

Use of membrane-based separation processes represents a promising alternative to traditional processes, since they do not require high energy and chemical consumption, thus significantly improving the sustainable energy approach [62,63]. In particular, the method based on the facilitated transport of organic and inorganic compounds across a liquid membrane (LM) is a powerful technology incorporating solvent extraction (SX) and stripping in a one-step continuously operating system. This method results in the significant reduction of solvent inventory requirement, volume of the contacting equipment, and cost, avoiding the production of by-products of difficult disposal [64,65,66].

A LM is a thin layer of an organic phase separating two aqueous solutions. This LM phase may also contain an extracting compound, indicated as carrier, which binds one or more components in the donor feed phase, transporting it (or them) from the donor phase (feed) to the acceptor phase (strip), resulting in the so-called facilitated transport. This approach responds to the process intensification strategy thanks to the benefit arising from the coupling of a chemical reaction and a mass transfer phenomenon.

LM-based processes possess numerous potential advantages with respect to conventional separation techniques, like the possibility to use expensive and highly selective extractants owing to their cyclical use, the high separation factors, the uphill concentration and separation, the minimization of chemical additive use, and the ability to separate low concentration species from very dilute solutions because of the effective binding.

Bulk liquid membrane (BLM), emulsion liquid membrane (ELM), and supported liquid membrane (SLM) are different types of LM-based systems. BLMs consist of two aqueous phases (feed and strip) separated by a water-immiscible liquid membrane phase. They are characterized by a small membrane surface area per unit volume, which makes them not attractive from a technological point of view [67,68]. EMLs are usually described as a bubble inside a bubble contained in the feed phase, where the inner bubble is the strip phase, closed by the LM membrane phase. ELMs are characterized by a large surface area per unit source phase volume, but are not technologically attractive, mainly because of the low emulsion stability [69,70].

SLMs generally consist of an organic solvent (the LM phase) contained in the pores of a hydrophobic microfiltration membrane and kept there by capillary forces [71]. SLMs have been tested in the separation and recovery of various metal ions [72,73,74,75], anions [76], molecules of biological interest, and organics [65,77,78] from different aqueous media.

### 3.2. Some Cases Studies on Application of Supported Liquid Membrane

Among the different metal ions, in 2019 Argurio et al. [72] investigated the recovery and concentration of neodymium from acidic media by extraction and transport across a traditional SLM. Neodymium was chosen as model rare earth element (REE). REEs play an important role in developing green and energy efficient technologies and high-tech industries. Due to the fast growth of technological applications of REEs, it is very important to develop techniques able to recover them selectively from aqueous media (e.g., acidic leaching solutions coming from hydrometallurgical treatment of waste electrical and electronic equipment). Two different carriers were compared in terms of operating pHs and extraction/transport performances. The results obtained during preliminary L-L extraction tests evidenced that D2EHPA (di 2 ethyl hexyl phosphoric acid) with respect to CYANEX 272 permitted to operate at lower pHs, both for the extraction and for the stripping operations. Thus, D2EHPA is indicated more for processing the very acidic aqueous leaching solutions coming from hydrometallurgical recovery of REEs from natural minerals and/or WEEEs (waste electrical and electronic equipment). An acceptable membrane permeability (0.381 cm min^−1^) and quite complete Nd recovery (99.14%) in the strip phase, corresponding to a feed/strip concentration ratio of 114, were obtained during the permeation test across the SLM. Despite the encouraging results, this work confirmed that the real Achille’s heel of SLM is represented by the low system stability, which was strongly influenced by the solubilization of the carrier in the aqueous phases.

**Figure 5:**
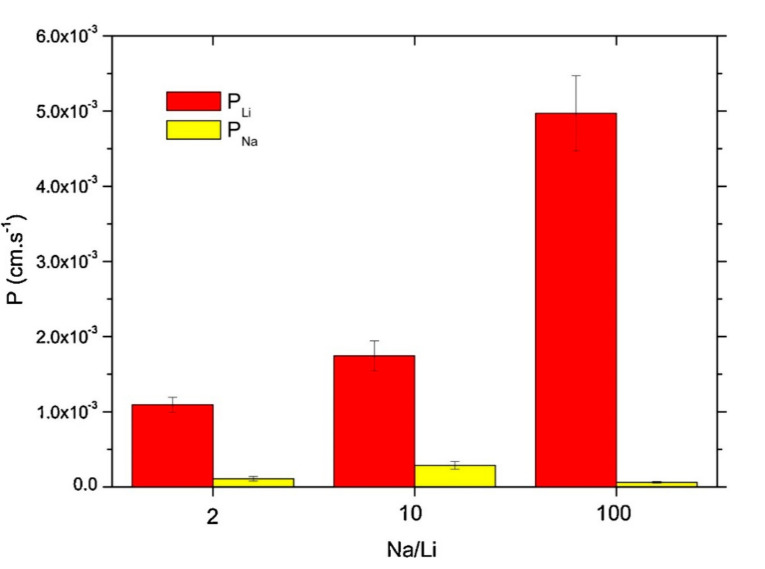
Lithium permeability (P_Li_) and sodium permeability (P_Na_) versus initial sodium/lithium molar ratio in the aqueous feed phase. Feed phase: LiCl (1 mmol L^−1^), NaCl (variable), pH = 12 [74].

An emerging and interesting application of SLMs is separation of lithium from sodium. The unique physicochemical properties of lithium (Li) makes this metal a good material to use for various applications, e.g., in the field of aerospace applications, in the glass and ceramics industry, and in automotive applications. Considering the latter applications, it is important to observe that lithium-ion batteries are the current solution to empower electric cars and to store energy, thanks to their high charge/weight ratio [79,80]. This consideration has a large impact on lithium consumption. Nowadays, the most important source of lithium is represented by lithium-rich brines coming from seawater desalination. Lithium extraction from this water remains challenging because of the high salinity, particularly due to the presence of sodium and potassium, with alkali metals having similar physicochemical properties to those of lithium. On this basis, the development of methods for selective recovery of lithium from lithium-rich brines represents an important research topic. Starting from this consideration, Zante et al. [74] demonstrated the possibility to applicate a supported liquid membrane (SLM) for the separation of lithium from highly concentrated sodium solutions with very high selectivity. Among various acidic extracting agents, heptafluoro-dimethyloctanedione (HFDOD) was the most efficient when used in synergistic combination with tri-n-octylphosphine oxide (TOPO) diluted in dodecane. As consequence of this synergy between HFDOD and TOPO, a high lithium extraction efficiency (>99%) and high selectivity for lithium over sodium (separation factor ca. 400) were obtained during preliminary solvent extraction tests. Lithium transport tests through SLM evidenced that high lithium permeability can be obtained even for high sodium/lithium concentration ratio, i.e., high sodium concentration and low lithium concentrations (Figure 5). A poor membrane stability was found, due to the leakage of the organic phase and the change in HFDOD:TOPO ratio as a result. Despite this limitation, which deserved due attention, it was shown that the SLM system is suitable for the extraction of lithium from low-concentrated solutions such as brines and seawater.

**Figure 6:**
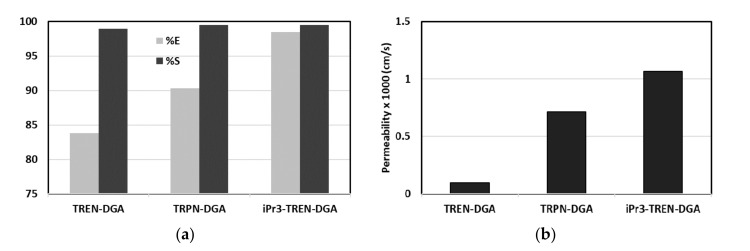
(**a**) Extraction, stripping, and (**b**) transport data of Am(III) from 3.0 M HNO_3_ aqueous phase using the three N-pivot tripodal diglycolamide (DGA) extractants (organic phase: 1.0 × 10^−3^ M extractant in 95% n-dodecane + 5% isodecanol) (data from [75]).

Separation of actinides from lean acidic effluents is one of the most challenging tasks in the radioactive waste management. Mahanty et al. [75] performed liquid–liquid extraction tests and subsequent SLM permeation tests for the transport of Am^3+^ ion from nitric acid feed solutions. Three N-pivot tripodal diglycolamide (DGA) ligands with varying substituent at the inner carboxamide N atom and spacer length were tested as the extracting agent. These ligands were indicated as iPr3-TREN-DGA, TREN-DGA, and TRPN-DGA. During metal extraction tests, the efficiency of the tested extracting agent was in the order iPr3-TREN-DGA > TRPN-DGA > TREN-DGA (Figure 6a). This trend was confirmed during the transport tests (Figure 6b), suggesting that the extraction was the limiting step. Instead, the diffusion of the complex across the SLM system was comparable for all the three extracting agents. Best membrane stability was obtained by using the TRPN-DGA ligand. The stability of the SLM was not satisfactory, suggesting that stabilization techniques, such as intermittent replenishment of the carrier solvent, are required to use the SLM system for the separation of Am from acidic feeds at large scale.

One important aspect to take into consideration when developing LM-based processes is the characteristic of used solvents, since the most widely used solvents are harmful and volatile. Thus, the academia and chemical industry are actively searching for alternative solvents. A possible class of solvents is represented by ionic liquids (ILs). ILs present several advantages over conventionally used organic membrane solvents, such as high thermal stability, negligible vapor pressure, low volatility, etc. Chasib et al. [81] studied the possibility to use room temperature ionic liquids (RTILs) as pure solvents as well as their binary mixtures as potential bulk liquid membranes (BLM) for extracting phenol and other phenolic compounds from industrial wastewaters. Different RTILs were compared in terms of their extraction and stripping performance. The results showed that a binary mixture ionic liquid (BMIL) membrane is better than single ionic liquid (SIL) membrane solvents. In particular, the binary mixture ionic liquid [Bmim] [(NTf_2_+PF_6_)], obtained by mixing 1-butyl-3-methylimidazolium bis(trifluoromethylsulfonyl) imide ([Bmim][NTf_2_]) and 1-butyl-3-methylimidazolium hexafluorophosphate [Bmim] [PF_6_], was the better one, since it provided high phenol stripping and extraction efficiencies with a minimal solvent loss and better stability in transport process.

### 3.3. Some Strategies Proposed in Literature to Improve LM System Stability

Despite the above mentioned advantages of LM techniques with respect to traditional ones and the encouraging results obtained by using SLM in the separation and recovery of different solutes from aqueous media, low stability of SLM remains a key issue for the large scale application of SLMs [72,74,76]. SLM instability is mainly due to the gradual loss of the LM phase out of the pores of the support, with its substitution with the aqueous solutions, thus creating water channels influencing both the flux and the selectivity [82,83].

Original solutions have been proposed by the scientists and engineers into the pertinent literature to enhance SLM stability. The simplest strategy, from a conceptual point of view, consists of the continuous regeneration of the LM by reimpregnation of the SLM when system performance degrades. By using this approach, Rehn et al. [84] proposed system regeneration by reimpregnation at 22–24 h intervals to maintain the stability of its hollow-fiber SLM. The results evidenced that the proposed approach permitted researchers to obtain the restoration of the SLM performance, but the aqueous feed and/or strip phases were polluted with the LM phase.

Another approach tested for stabilizing a SLM consisted of the use of the so-called feed and/or strip dispersion phases [85,86,87,88]. By using this technique, the increase of SLM stability, intended as maintenance of operating performance, was obtained by circulating, as feed and/or strip phase, a mixture of the aqueous feed and/or strip and organic LM phase, continuously stirred in order to contain uniformly dispersed organic droplets. This approach permits users to maintain system performance for a longer time, but presents two important limitations. First of all, the loss of the LM phase is not blocked, thus resulting in contamination of the aqueous phases with the organic LM phase. Secondly, by operating in this way the volume of the LM phase is not minimized—that is one of the reasons for developing SLM instead of classical L-L extraction.

The creation of protective layers that limit the loss of the organic LM phase from the pores of the membrane support can also increase the stability of the SLM system. Some approaches based on this idea are interfacial polymerization or plasma polymerization surface coating [89,90]. By using this approach, Yang et al. [89] modified a hydrophobic microporous microfiltration membrane with pore sizes of 0.05–0.2 μm by using hexamethyldisiloxane and heptylamine as polymerizing agents. The results evidenced that the stability of the SLM in copper transport using LIX984 as the carrier was improved, but a reduced permeation flux was obtained.

A conceptually similar approach that gave encouraging results in terms of SLM stabilization is represented by the so-called total or partial gelation of the LM phase [91]. Using this approach, in 2018, Ren et al. [92] prepared, via gelation technique, an extraction gel membrane (EGM). In the EGM, an extraction gel layer was formed on the shell side of PVDF ultrafiltration hollow fibers. The results evidenced that the proposed approach permitted them to avoid the loss of the carrier from the LM phase. On the basis of this enhancement, both good permeability and increased system stability were obtained. Despite these encouraging results, some important factors have to be properly evaluated: (i) reproducibility of membrane preparation; (ii) suitability of membrane coating with a thin extraction gel layer for practical purpose; (iii) precise control of the interface between the aqueous phases and LM, which is required but technically challenging [93].

Other interesting and promising approaches proposed by scientists and engineers for increasing SLM stability start from the idea of designing different SLM configurations for avoiding/minimizing the release of the LM phase in the adjacent aqueous phases. A potential promising approach is based on the use of membrane contactors, where solid membranes mediate the contact between the organic and the aqueous feed and strip phases.

**Figure 7:**
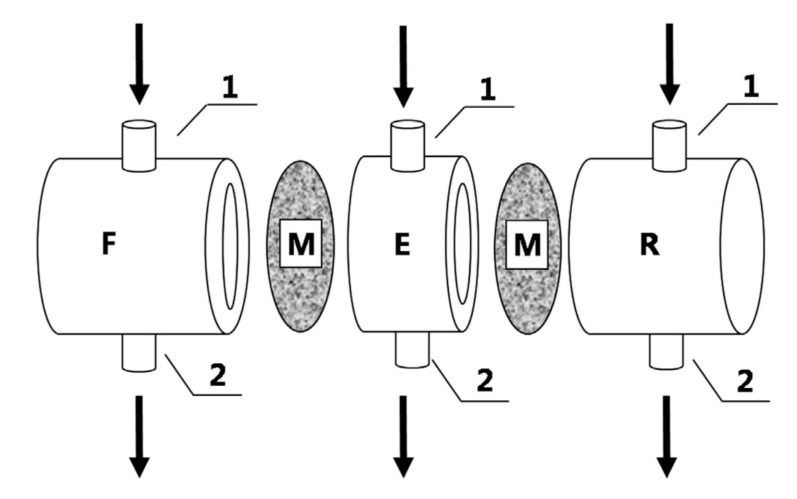
Schematization of flowing liquid membrane system. F, E, and R: feed, organic, and strip compartments; M: separation membranes. 1 and 2: inlet and outlet of the feed, organic, and strip solutions [97].

Contained liquid membranes and flowing liquid membranes represent two similar approaches, with an important difference. By using contained liquid membranes, the stability of membrane system was improved, but the organic phase was static and the permeation of solutes from feed to strip phase was a pure diffusion [94,95]. In flowing liquid membranes, the LM phase flows in a chamber limited by two solid membranes, thus reducing the mass transfer resistance [96,97]. In these systems, the solid membranes are permeable to solutes, thus permitting their permeation across the SLM system (Figure 7). The loss of the LM phase into the adjacent aqueous phases is avoided by the solid membranes, acting as a barrier to LM phase loss. A significant stability enhancement was obtained by operating with this configuration, but the obtained fluxes were lower with respect to the traditional SLM configuration, because of the higher transport resistance.

An interesting system, named hollow fiber nondispersive solvent extraction (NDSX) and stripping, was recently considered by some research groups. The organic LM phase is continuously circulated across two hollow fiber modules operated as membrane contactors. Considering the LM phase circulation, it can be affirmed that this kind of system also belongs to the category of flowing liquid membranes. Based on this approach Galan et al. [98] studied the separation of nickel and cadmium from highly concentrated solutions. In the proposed system the extraction and stripping processes were performed simultaneously in two parallel modules, where the organic phase was continuously recirculated in a closed circuit. The results evidenced that under the considered experimental conditions, a constant rate of mass transport was obtained, leading to a selectivity factor in the concentration strip s = 67 mol Cd/mol Ni. The main limitation of this approach consists of the high volume of organic phase involved.

The same nondispersive solvent extraction (NDSX) approach was used by Raut and Mohapatra [99] for separating uranium from nitric acid solutions using three dialkyl amides (indicated as DHHA, DHOA, and DHDA) both alone and in mixture with TODGA (N,N,N’,N’-tetra-n-octyldiglycolamide) in normal paraffinic hydrocarbon (NPH) as the carrier solvents. In this study, the same commercial hollow fiber contactor, operated in recirculation mode at a flow rate of 3 L h^−1^, was used for both extraction and stripping experiments. A first set of experiments were carried out in order to study the extraction uranium from nitric acid solutions. The stripping of uranium from the loaded organic phase was carried out in a separate set of experiments, using 0.01M HNO_3_ or 1M Na_2_CO_3_ as the stripping agent. The results evidenced that the use of TODGA with the amides permitted researchers to obtain a faster extraction and stripping higher efficiencies. In the extraction tests, about 90% of uranium was extracted within 60 min. In the stripping tests, 90% recovery of uranium from loaded organic phase was achieved using 1M Na_2_CO_3_ as stripping agent.

**Figure 8:**
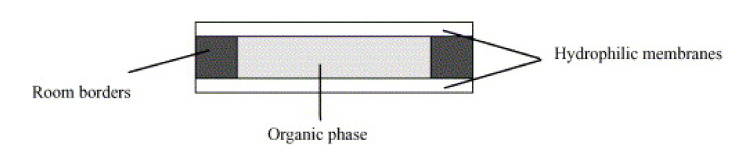
Schematic representation of the stagnant sandwich liquid membranes (SSwLM) [71].

Starting from the limitation of flowing liquid membranes, Molinari et al. proposed stagnant sandwich liquid membranes (SSwLMs) [15]. SSwLMs were assembled by sandwiching the organic LM phase between two thin hydrophilic membrane supports (Figure 8). In this system, a very thin film of the LM phase was sandwiched and situated between the two aqueous feed and strip phases. The functioning of this system was demonstrated in the separation of both organic species [65,100] and metal ions [64,71] from aqueous media. The results evidenced that SSwLMs permitted researchers to increase system stability (14 times higher in the case of gemfibrozil), since the two hydrophilic membrane supports acted as a barrier to LM phase loss, and to increase the permeation flux (two times in the case of gemfibrozil), since the transport resistance was decreased with respect to that one of traditional SLM.

## 4. HMPs Obtained by Coupling Membrane Processes with Water Soluble Complexing Agents

### 4.1. Foundamentals of Complexation–Ultrafiltration

Many types of industrial wastewaters, such as the ones coming from mining, mineral processing, metal finishing, electroplating, and battery industries, contain high concentrations of heavy metals as multicomponent mixtures [101,102,103]. As previously reported, some traditional methods are available to separate/recover metal ions from contaminated waters, but these techniques, while respecting the legislative limits, have numerous limitations [15]. Thus, the development of innovative processes with high performances and low environmental impact is crucial for a sustainable development.

**Figure 9:**
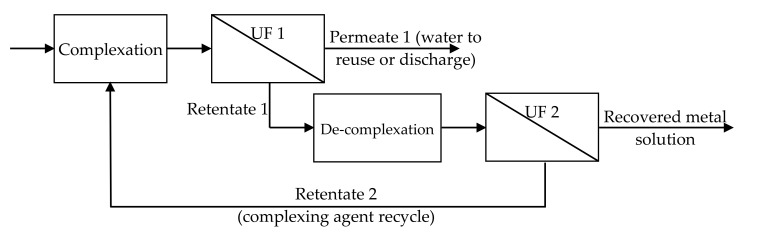
Schematization of the overall complexation–ultrafiltration (CP–UF) process.

Among the different environmentally friendly processes for heavy metal removals from aqueous media, complexation–ultrafiltration (CP–UF) represents a promising technique [104,105,106]. The starting point of this technique was the idea of using membrane processes to remove these pollutants from waters. Considering the low molecular dimension of heavy metals, reverse osmosis membranes should be required for their removal, resulting in high operative costs, low permeate flow rates, and low selectivity. These limitations can be overcome by considering that heavy metal ions can be retained by an ultrafiltration membrane, if they are preliminarily bounded to a water-soluble complexing agent. This complexation step is followed by an ultrafiltration step, where the complexing agent and its complexes are retained by the membrane, while the permeate is the purified water, which can be reused or safely discharged (Figure 9) [107,108]. In order to make sustainable this technique, the recovery and reuse of the complexing agent is required. Considering that the complexation process is influenced by chemical parameters (e.g., the pH), the complexing agent is recovered by releasing the metal, e.g., by decreasing the pH of the retentate coming from the first ultrafiltration step. This decomplexation step is followed by another UF step. The retentate obtained by this filtration step is a complexing agent-rich phase, which can be recycled at the decomplexation step, while the permeate is a concentrate solution of metal salts that can be reused in the industrial process. The overall process can be schematized as reported in Figure 9.

If the complexing agent is a polymer, this technique is also indicated as polymer-assisted ultrafiltration (PAUF).

When operated as reported in Figure 9, the CP–UF process can achieve the recovery and recycle of heavy metals and the reuse of polymers, which has huge economic and environmental benefits. On this basis it can by affirmed that the CP–UF process opens up a new way for the treatment of wastewater contaminated by heavy metals.

### 4.2. Selective Separations by Complexation–Ultrafiltration

One important advantage of the CP–UF technique is the possibility to achieve high selectivity by using a proper selective binding agent. This selectivity can be due to the chemical properties of the complexing agent, allowing it to complex one specific metal ion, and/or to the opportune choice of the operating conditions [109,110]. The complexing agent/metal weight ratio and the operating pH are the main operating parameters that affect the complexation step. An appropriate choice of these parameters can be used for obtaining selective separation, mainly because the pH range of a polymer–metal complex formation varies significantly from metal to metal. Starting from this consideration Molinari et al. [104] performed the selective separation of Ni(II) ion from Cu(II) ion, both contained in a same solution, by CP–UF, using polyethylenimine (PEI) as the water-soluble polymer and two UF membranes with different cut-offs as the separation medium. Preliminary complexation tests were carried out in order to determine the optimal operating conditions for copper and nickel complexation and decomplexation by the PEI polymer. The results evidenced that the optimal chemical conditions were pH 6.0 and 8.0 and polymer/metal weight ratio of 3.0 and 6.0 for copper and nickel, respectively. On the basis of these results, UF tests were carried out by changing the operating pH, the polymer/metal weight ratio, the transmembrane pressure (TMP), and the membrane cut-off. It was evidenced that by operating at pH 6.0 (value for obtaining preferential copper complexation) and a polymer/metal weight ratio of 3.0 (value needed to bound only copper ions), a high process selectivity was obtained. In particular, copper ion, preferentially complexed by the polymer, was recovered in the retentate (94% recovery), while nickel ion was recovered in the permeate (100% recovery). The selectivity of the CP–UF was tested by using as aqueous matrix both distilled water and a torrent water as a real aqueous effluent. Similar results were obtained in terms of metals recovery, but a higher membrane fouling was obtained by using the torrent water.

One year later Zeng et al. [110] tested the possibility to obtain the selective separation of Hg(II) from Cd(II) by the CP–UF process. The water-soluble polymer poly (acrylic acid) sodium salt was used as the complexing agent. Process selectivity was calculated as follows:

β_(Cd/Hg)_ = (1 − R_Cd_)/(1 − R_Hg_) (1)

where β_(Cd/Hg)_ is process selectivity, and R_Cd_ and R_Hg_ are Cd(II) and Hg(II) rejection coefficients, respectively.

The effect of some operating parameters on process selectivity has been examined. In particular, obtained results evidenced that loading rate, pH, concentration of salt added, and low-molecular competitive complexing agents affect significantly process selectivity. Thus, it is important to use optimal operating conditions (metals/polymer weight ratio 1.5 and pH 5) to obtain high process selectivity. UF tests were carried out under optimal conditions, obtaining a 100% Hg rejection, in contrast with a 10% Cd rejection. As a consequence, a rapid linear increase of mercury concentration and a very slow increase of cadmium concentration in the retentate were obtained. The so obtained retentate was submitted to a diafiltration test to separate further both metals. The results evidenced a sharp decrease of Cd concentration in the retentate, whereas mercury concentration remained always constant. Then, the diafiltrate was a solution containing a large quantity of Hg(II) and very little amount of Cd(II).

### 4.3. Stability of Polymer–Metal Complex in the Shear Field: A Possible Strategy for Achieving Both Polymer Regeneration and Separation Selectivity

**Figure 10:**
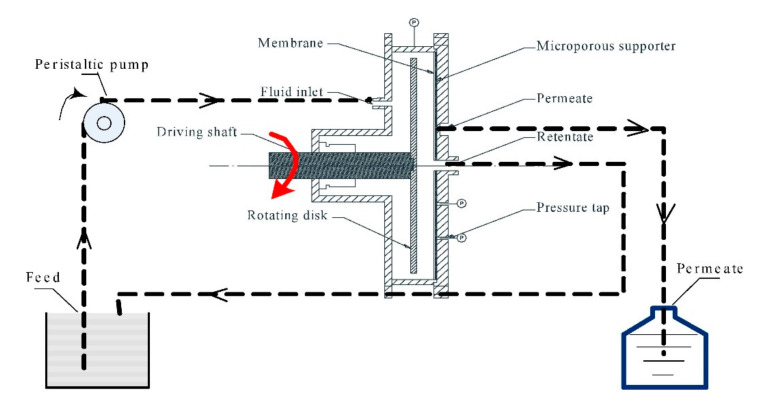
Schematization of the rotating disk enhancing CP–UF process [105].

Despite its potentialities, the complexation–ultrafiltration technology has not yet been applied in industry. One of the key bottleneck problems in the industrial application of CP–UF is due to the loss of polymer–metal complex stability when the shear rate exceeds the critical shear rate [105]. Gao et al. [105] reported that when they treated heavy metal wastewater with hollow fiber membrane, very different heavy metal rejections were obtained by using different flow-making equipment (peristaltic pump and centrifugal pump). This trend was ascribed to the instability of the polymer/metal complex under the high shear rate produced by the high rotating speed of the centrifugal pump, resulting in the dissociation of free metal ions and the reduction of the metal rejection. Thus, the study of the stability of the polymer–metal complex in the shear field is of great significance for the industrial application of CP–UF. Starting from these considerations, the authors tested the CP–UF process for treating wastewater containing nickel using sodium polyacrylate as polymeric complexing agent. A rotating disk membrane module was used (Figure 10) to generate a shear field on the membrane surface, thus evaluating the influence of the shear field on the stability of polymer-Ni complex. The results evidenced that Ni^2+^ ions can be removed with a rejection of 98.26% by operating at neutral pH (7.0), polymer/Ni weight ratio of 13 by rotating the disk at a rotating speed lower than 848 rpm. When the rotating disk was operated at a velocity higher than 848 rpm, the high shear rate produced by the rotating disk caused the dissociation of the polymer-Ni. In these conditions, the recycling of Ni^2+^ (recovered into the permeate) and the reuse of the polymer (recovered into the retentate) can be achieved, which has huge economic and environmental benefits. On the basis of their results, the authors also affirmed that the influence of the shear field on the stability of polymer–metal complex can be useful to obtain a good process selectivity. Indeed, considering the shear stability difference of polymer-heavy metal complexes, the selective separation of heavy metals can be obtained by appropriately modulating the rotating velocity.

By using the same rotating membrane disk, the same research group also studied the removal of copper(II) ions from aqueous solutions by CP–UF, giving particular attention to the shear stability of polymer–Cu complex [111]. The results evidenced that the rejection of Cu(II) could reach 99.6% at pH 6.0 and polymer/metal weight ratio 25. The authors also evidenced that the determination of the shear stability and of the critical shear rate of the polymer–metal complex can give guidance to the selection of the delivery pumps so that high removal efficiency can be obtained in the industrial application of complexation–ultrafiltration. In addition, shear-induced dissociation and ultrafiltration can be used to regenerate the polymer.

The same approach was used one year later to perform the removal of Cd (II) from dilute aqueous solutions by CP–UF and to study the shear stability of polymer–Cd complex [112]. Two different rotating disks, disk I (without vane) and disk II (with six rectangular vanes) were used. A satisfactory rejection (99.7%) was obtained at rotating speed lower than 2370 and 1320 rpm for disk I and disk II, respectively. However, when rotating speed exceeded these values, the rejection of Cd (II) decreased greatly. Also, in this study the authors evidenced as the limited shear stability of polymer–metal complex can be used to induce dissociation and then polymer regeneration.

These works [105,111,112] evidenced a problem that limits the applicability at large scale of CP–UF, but, if opportunely analyzed, it can be controlled and even used to obtain polymer regeneration and/or to control polymer selectivity, which has huge economic and environmental benefits.

### 4.4. Influence of Membrane Characteristic on CP–UF Performance: A Case Study.

It can be easily understood that the characteristic of the membrane (i.e., membrane pore size, membrane porosity, membrane hydrophilic/hydrophobic character, water swelling, water permeability) strongly affect the performance of the CP–UF process. Thus, it is also important to use a membrane with adequate characteristics in order to obtain satisfactory separation/recovery. On this aspect, in a very recent article, Nafti Mateur et al. [113] proposed an innovative method to prepare porous membranes with controlled porosity for filtration purpose. For preparing the membrane, the authors proposed the use of hexagonal boron nitride nanosheets (h-BNNS), to stabilize microdroplets of castor oil in a continuous homogeneous gelatin solution. The acquired experience evidenced as, by using the proposed approach, the porous structure (i.e., membrane pore size, hydrophobicity) of the prepared membrane was strongly influenced by two factors: (i) the duration of the drying after emulsion casting and (ii) the duration of the cross-linking step. A difference in the porous structure had a significant influence on water permeability. By controlling these parameters, a membrane with pore size between 0.39 and 1.60 μm and displaying water permeability between 150 and 506 L h^−1^ m^−2^ bar^−1^ was obtained. This membrane was tested for removing iron ions from aqueous solution by CP–UF, by using poly (acrylic acid) (PAA) as the polymeric binding agent. A high removal rate (97%) was obtained at pH ≥ 5 with three bars of transmembrane pressure.

## 5. HMPs Obtained by Coupling PMRs and CP–UF Processes

An example of the integration of more hybrid processes is the removal of arsenic from waters. Having observed that As(V) is complexed more easily than As(III), the photocatalytic oxidation of As(III) to As(V) was followed by the CP–UF process. In the following, more details are given.

The arsenic purification from water is a very important environmental problem because the contamination of ground and surface water by arsenic can cause health problems to many people if there is an excessive arsenic amount in the contaminated drinking water [114,115]. For this purpose, Molinari et al. studied the application of CP–UF in the separation of arsenic ions from water [116].

As before described, the CP–UF technique is mainly used to bind metallic ions [116,117], through a complexation step that is followed by a UF step. During this UF step the recovery of the polymer is very important, in terms of process sustainability, which is attained by releasing the metal from the polymer metal complex, e.g., by decreasing the pH of the solution and subsequent UF [118,119].

The first step to obtain an efficient As removal process is the As(III) oxidation into the more easily extractable As(V) form [120]. The oxidation procedure can be performed in different ways but a most interesting and sustainable alternative for oxidizing As(III) to As(V) is the photocatalysis (PC) [121,122,123]. For this reason, Molinari et al. in their work combined the complexation–ultrafiltration (CP–UF) technique and the photocatalytic process, showing that this combination permits users to obtain a quite complete arsenic removal from the treated water [116].

Ultrafiltration tests were carried out in a batch system [124], operated in dead-end mode. It was constituted by two cylindrical stirred cells in clear polycarbonate (model A-02910-41 from Cole-Parmer International, Vernon Hills, IL, USA) with a maximum chamber capacity of 72 mL. The membrane used had a diameter of 43 mm, with an exposed membrane surface area of 14.5 cm^2^. To reduce fouling problems related to polymer accumulation on the membrane surface, a magnetic stirring bar, controlled by a magnetic stirrer, was used. All the diafiltration tests were carried out at room temperature (25 ± 1 °C) with a controlled pressure of nitrogen.

Experimental photocatalytic oxidation of As(III) to As(V) was carried out in the set-up that was constituted by a cylindrical Pyrex glass reactor with a volume of 500 mL and with three outputs in its top section for sampling the suspension and bubbling oxygen in the reacting mixture. The lamp (125 W immersed medium pressure Hg lamp that emitted mainly in the UV region) was positioned axially inside the reactor with a Pyrex glass jacket that maintained the system at a temperature of 30 °C by circulation of thermostatically controlled water. The results showed that the photocatalytic oxidation of As(III) was successfully performed under UV radiation by using TiO_2_ (0.05 mg L^−1^) as the photocatalyst, O_2_ as the oxidant, and pH 9. For the CP–UF step, the authors tested two polymers: poly(dimethylamine-coepichlorohydrin-coethylenediamine) (PDEHED) and poly(diallyl dimethyl amnmonium chloride) (Poly-DADMAC). The best performance was shown by the polymer PDEHED, in the pH range of 7–8, while at higher pH the PolyDADMAC was better. In this work, the author obtained a quite complete arsenic removal from the treated water by coupling the photocatalytic oxidation of As(III) to As(V) and the CP–UF process. It was observed that the performance of the CP–UF step could be enhanced by using a membrane with lower MWCO, higher polymer amount, and/or polymer prefiltration. Moreover, the performance of the photocatalytic step during the photo-oxidation process can be increased by using a higher photocatalyst amount and a better pH control.

**Figure 11:**
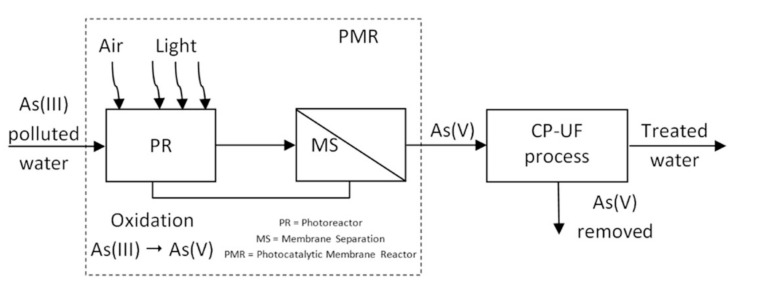
Conceptual coupling of photocatalysis and CP–UF (PR = photoreactor, MS = membrane separation; PMR = photocatalytic membrane reactor) [116].

Furthermore, the authors reported a conceptual scheme of the photocatalysis and CP–UF coupling and some criteria to operate the CP–UF process (Figure 11). The photocatalytic step, carried out in the photoreactor previously mentioned, can be efficiently used to oxidize As(III) to As(V). The CP–UF process includes two steps: (i) complexation of As(V), that is the target species in this work, to the polymer until getting the maximum loading capacity and (ii) the decomplexation to regenerate and to recycle the polymer. To load the polymer, the method that can be used is the diafiltration in continuous (DF-c), with the continuous feeding of the aqueous solutions containing the pollutant. To regenerate the polymer, instead, the diafiltration can be useful with volume reduction (DF-vr) and its conditioning to the optimum pH.

## 6. Other Hybrid Membrane Processes

Considering the continuous attention in water resources limitations and the necessity of water reuse as a strategy to overcome the water demand, the situation of water use in the oil refining industry is another case of interest. Indeed, it is estimated that large oil refineries consume approximately 1 m^3^ of fresh water per 1 m^3^ of processed oil. Then, a large volume of effluent is produced. This effluent, adequately treated, can be reused making the refining process more sustainable. A possible process is represented by a membrane bioreactor, in which the bio-degradation process is coupled to a filtration on micro or ultrafiltration membrane.

Considering the specificity of the refining industry, an improved configuration, called osmotic membrane bioreactor (OMBR), can be useful. The main advantages of OMBR with respect to conventional MBR are its low fouling propensity, as it does not require a hydraulic pressure for water permeation, and high removal efficiency of low molecular weight compounds, which ensures a good quality for the permeate. Despite these features, OMBR undergoes salinity build up because of the high rejection of salts by RO membrane, in addition to the reverse salt flux from the draw solution (DS). Increased salinity in the biological system can lead to higher membrane biofouling. Besides, salts precipitation onto the membrane surface may be also pronounced. To mitigate these effects, a UF or MF membrane can be added in the bioreactor so that the porous membrane removes inorganic salts and other dissolved constituents. The addition of UF-OMBR gives satisfactory results for those applications that do not require a high physicochemical quality for the water reuse. For more complex effluents, containing also slow degradable and/or recalcitrant compounds, the build-up of recalcitrant compounds must also be considered. In this context, Barbosa Moser et al. [125] assessed the impact of two different draw solutions (DS) on salinity and organic build-up in a hybrid ultrafiltration-osmotic membrane bioreactor (UF-OMBR) treating oil refinery effluent. The salt contained into the two DS were NaCl and CH_3_COONa and the performance of the system’s main parameters were analyzed in terms of permeate flux, stability of the biological community, organic matter and nitrogen removal, and membrane fouling. The use of a biodegradable salt as CH_3_COONa, in addition to preventing the salinity accumulation, should promote better conditions for the biological process giving a gain in the removal of recalcitrant compounds. The operation was conducted continuously for 505 days.

The results evidenced that by using NaCl in the DS solution, the UF was not able to avoid recalcitrant build up in the reactor, which resulted in a progressive decrease of process efficiency. The use of CH_3_COONa as salt in the DS favored the microbiological activity and enhanced recalcitrant biodegradation. Despite this good result, the presence of the biodegradable compound CH_3_COONa favored the microbial growth over the membrane thus leading to a biofouling layer formation. As a consequence, a lower permeate flux (0.60 ± 0.15 L m^−2^ h^−1^ vs. 1.07 ± 0.32 L m^−2^ h^−1^) occurred when CH_3_COONa was used. The overall result of this work evidenced as, in view of the higher DOC removal, the UF-OMBR operation with CH_3_COONa represents a viable option for refinery wastewater treatment and recalcitrant compounds biodegradation.

In recent years, wastewater treatment plants (WWTPs) have become diffusers of some emerging contaminants (ECs) into the environment since conventional processes are not efficient in their removal. Based on this, the integration of advanced treatment methods in the WWTPs is very essential. One of the most important challenge when integrating tertiary treatment after biological processes for the removal of ECs is due to the presence of natural organic matter (NOM). By using membrane processes as post-treatment of the biological process, an important membrane-fouling was promoted by NOM, thus affecting process efficiency and limiting the applicability of membrane processes. By using the adsorption process, NOM competes with ECs for adsorption sites, thus increasing the required amount of adsorbent. Starting from these considerations, Naddeo et al. [126] proposed an innovative hybrid membrane process called USAMe^®^, which integrates ultrasound irradiation (US), adsorption (A), and membrane filtration (Me) for the removal of ECs from secondary effluents. Three ECs were considered: diclofenac, carbamazepine, and amoxicillin. These ECs were selected because of their large rates of consumption and frequent presence in the aquatic environment. These three ECs were spiked into a real secondary wastewater effluent at two concentrations: 10 ppm and 100 ppb, thus evaluating the ability of the proposed process to treat effluent polluted at ppm and ppb level. Membrane ultrafiltration and its combination with US (USMe) or adsorption (AMe) were also studied as control tests. The results evidenced that the combination of all the three methods in the USAMe^®^ hybrid process minimized the unfavorable effects of NOM observed in the individual methods, permitting researchers to obtain an EC removal of 99%, higher than that one obtained by the AMe alternative (90%). The satisfactory EC removal for both ECs and NOM, with its continuous cleaning action, makes USAMe^®^ an ideal hybrid membrane process (HMP) applied after biological process. Moreover, the results evidenced that the USAMe^®^ process was efficient not only in the removal of ECs from secondary effluents, but also in generating safe and less toxic treated effluents; thereby displaying its potential as an advanced method for wastewater treatment.

Despite these very interesting results, as also emphasized by the authors, the effectiveness of the hybrid USAMe^®^ process could be further enhanced through experiments on the treatment of wastewater containing lower EC concentrations. Besides, a deeper investigation of intermediates and transformation products is required. Design improvements, alternative membrane materials, as well as modes of ultrasound application for efficiency and economy purposes also need to be explored.

## 7. Perspectives/Future Directions

The described HMPs coupling membrane separation and/or biological or chemical reaction can play a key role when pollutants contained in wastewater must be removed (destroyed by degradation or recovered). In Table 1, the summary of the various applications described in the present work are reported. They must be considered not exhaustive and can be used as examples to ideate/design new coupling and opportunities where HMPs can make a breakthrough compared to the previous processes. For each industrial sector a dip study of the process, the knowledge of problems that must be solved and the study of existing literature can give the basis to design a process innovation. However, it must be considered that in many cases innovation takes too much time because companies are oriented to invest after proof of process robustness. This means designing and testing a suitable pilot plant before deciding a significant capital investment.

The examples reported in Table 1 and described in this review evidence the variety of combinations that can be made to build an HMP and that it is very important to appropriately develop and conduct these processes. In particular, in the case of PMRs, it is necessary to adopt/design useful strategies to reduce membrane fouling effects (e.g., bubbleless backpulsing or other methods). In the case of an LM-based process, is important to take into consideration system stability, which can be increased by developing appropriate SLM configuration to avoid/minimize the release of the LM phase in the adjacent aqueous phases, which is the main cause of system instability.

The sustainable use of the polymer and process selectivity represents key issues in the CP–UF process. Thus, it is important to determine appropriately the operative conditions for complexation and decomplexation and to design appropriate set-up to conduct the overall process. The results discussed in the present paper evidenced that a satisfactory system selectivity can be obtained in a “simple” way by appropriately fixing the operating conditions, i.e., pH and complexing agent/metal ratio.

Future direction in the development of HPMs requires an accurate design of the overall system and an accurate choice of the operating conditions. Furthermore, membrane materials and modules, the development of materials permitting the use of renewable sources, and the transfer of the results from laboratory to large-scale applications also need to be explored.

## 8. Conclusions

In this review paper, the feasibility of hybrid membrane processes (HMPs) for advanced wastewater treatment has been shown, describing some HMPs and related applications.

The HMPs considered were: (i) photocatalytic membrane reactors (PMRs); (ii) liquid membrane (LM)-based systems; (iii) complexation–ultrafiltration (CP–UF); (iv) CP–UF coupled with PMR, which is a more complicated hybrid system; (v) HMPs not belonging to the previous categories, such as osmotic membrane bioreactor (OMBR) and the innovative hybrid membrane process called USAMe^®^.

Application of these HPMs for obtaining reclaimed wastewater could represent an advanced method for producing a suitable quality water resource for non-potable or (indirect) potable use. HMPs, improve the potentialities of classical treatment methods and those of membrane separations, giving a synergistic effect, which minimizes the economic and environmental impacts. Thus, it can be affirmed that HMPs represent a promising approach in terms of overall sustainability, which must be the mission of recent world development.

## Figures and Tables

**Table 1:** Summary of applications, types of coupled processes in the hybrid membrane processes (HMP), and main advantages.

Application	Types of Coupled Processes in the HMP	Main Advantages	Ref
Removal of secondary effluent organic matter (SEOM) from secondary effluents (SE)	Photocatalytic membrane reactor (PMR)	SEOM degradation > 60%reduction of membrane fouling propensity	[30]
Treatment of primary (PE) and secondary effluents (SE) coming from municipal wastewater treatment plant	PMR with UF and direct contact membrane distillation (DCMD)	The coupling photocatalysis-membrane processes resulted in increased the permeate flux only in the case of UF	[31]
Treatment of SE coming from a municipal wastewater treatment plant for removing pharmaceuticals	PMR coupling UF with: UVC/H_2_O_2_ UVC/TiO_2_ UVC	100% OTC removal 49% mineralization in 5 h in the UVC/TiO_2_-UF system	[35]
Photocatalytic degradation of red blue 5 (RB5)	PMR	88.89% RB5 degradation by using ZnO in 180 min 98.34% by using Fe^3+^@ZnO in 180 min	[44]
Photocatalytic degradation of rhodamine B (RhB)	PMR	RhB degradation > 60% increased antifouling ability using a visible-light-driven g-C_3_N_4_/TNA membrane	[45]
Photocatalytic degradation of methylene blue (MB)	PMR	MB degradation > 90% for four consecutive runs using P-doped g-C3N4 (PCN) as photocatalyst	[46]
Photocatalytic degradation of MB	PMR	90% MB degradation after 120 min under visible light by using Ag/GO/TNTs	[48]
Photocatalytic degradation of MB	PMR	83.5% MB degradation under simulated solar light irradiation	[37]
Photocatalytic degradation of lanasol blue 3R (LB)e	PMR	up to 91.42% LB degradation also after 5 degradation cycles excellent self-cleaning ability using the modified (TiO_2_/PSS) membrane showed	[50]
Photocatalytic degradation of bisphenol A (BPA)	PMR	90% BPA removal under UV with dual-layer hollow fiber membrane (DLHF) 81.6% BPA removal under visible light using the N-doped TiO_2_ DLHF	[56]
Photocatalytic degradation of diclofenac (DCF)	Submerged PMR (SMPR) with suspended photocatalyst	Pseudo-first-order kinetic Beneficial effect of H_2_O_2_ of on system performance	[59]
Photocatalytic degradation of paracetamol (PCT), furosemide (FRS), nimesulide (NMD), diazepam (DZP)	Tube-in-tube PMR	27.4% (PCT), 35.0% (FRS), 24.2% (NMD) and 30.0% (DZP) degradation in synthetic wastewater at steady-state for the UVC/H_2_O_2_/TiO_2_ system. Lower degradations by using an urban wastewater after secondary treatment	[61]
Recovery and concentration of neodymium from acidic media	Supported liquid membrane (SLM)	0.381 cm min^−1^ membrane permeability 99.14% Nd recovery 114 feed/strip concentration ratio	[72]
Selective recovery of lithium from lithium-rich brines	SLM	High selectivity over lithium even for high sodium/lithium concentration ratio	[74]
Separation of actinides from lean acidic effluents	SLM	ca. 98% extraction ca. 99% stripping	[75]
Selective separation of Ni(II) from Cu(II)	CP–UF	94% Cu(II) recovery in retentate 100% Ni(II) recovery in permeate Same selectivity but a higher membrane fouling by using a real aqueous effluent	[104]
Selective separation of Hg(II) from Cd(II)	CP–UF	100% Hg rejection 10% Cd rejection. Diafiltration of the CP–UF retentate permitted increase separation selectivity	[110]
Recovery of nickel from wastewaters	CP–UF in a rotating disk system	98.26% Ni^2+^ rejection at a rotating speed RS < 848 rpm polymer regeneration and nickel recovery at a RS > 848 rpm	[105]
Removal of copper(II) from aqueous solutions	CP–UF in a rotating disk system	99.6% Cu(II) rejection. Shear stability and critical shear rate of the polymer–metal complex are important in view of industrial applications of CP–UF polymer regeneration and copper recovery at a RS > critical RS	[111]
Removal of arsenic ions from water	CP–UF coupled with photocatalysis	Quite complete As removal by coupling photocatalytic oxidation of As(III) to As(V) and CP–UF	[116]
Treatment of oil refinery effluent	Hybrid ultrafiltration-osmotic membrane bioreactor (UF-OMBR)	The use of CH_3_COONa instead of NaCl as salt in the draw solution (DS): favored the microbiological activity enhanced recalcitrant biodegradation favored the microbial growth over the membrane, thus leading to a biofouling layer formation with consequent decrease of flux (0.60 vs. 1.07 L m^−2^ h^−1^)	[125]
Removal of emerging contaminants (ECs) from SE	HMP called USAMe^®^, ultrasound irradiation (US), adsorption (A), membrane filtration (Me)	ECs removal of 99% by using the USAMe HMP.	[126]
